# Genetic correlation analysis of calving ease and gestation length of Korean Holstein cattle

**DOI:** 10.5713/ab.24.0431

**Published:** 2025-01-15

**Authors:** Mahboob Alam, Jae-Gu Lee, Chang-Gwon Dang, Seung-Soo Lee, Sang-Min Lee, Ha-Seung Seong, Mina Park, JaeBeom Cha, Eun-Ho Kim, Hyungjun Song, Seokhyun Lee, Joonho Lee

**Affiliations:** 1 Animal Breeding and Genetics Division, National Institute of Animal Science, Rural Development Administration, Cheonan, Korea; 2 Dairy Cattle Improvement Center of NH-Agree Business Group, National Agricultural Cooperative Federation, Goyang, Korea; 3 GENEAPPS, Seoul, Korea

**Keywords:** Calving Ease, Direct Effect, Genetic Correlation, Gestation Length, Korean Holstein, Maternal Effect

## Abstract

**Objective:**

To investigate genetic correlation between calving ease (CE) and gestation length (GL) traits of Korean Holstein cattle to understand genetic structures of these two traits and their potential implications.

**Methods:**

Records of progenies from first parity (P1, N = 117,921) and second parity (P2, N = 141,104) Holsteins cows were used for analysis. All phenotypes (CE and GL) were considered as calf traits. The CE was an ordered categorical trait. It was scored from 1 (normal calving) to 4 (difficult calving). GL observations were restricted between 260 and 305 days. Variance components and genetic parameters were estimated through a bivariate animal model with a correlated maternal effect using the BLUPF90+ software package.

**Results:**

Heritability (h^2^) estimates of CE for direct and maternal effects were low (less than 0.01) in all parity calves. For GL, despite lower h^2^ of maternal effect (~0.03), the direct effect was moderately heritable (0.20 to 0.23) in this study. Direct and maternal effects of CE trait were weakly correlated (P1: 0.09±16.60, P2: −0.04±0.00). GL had similar correlation patterns (P1: 0.03±0.00; P2: −0.15±0.05) across parities. Direct genetic correlations of GL and CE were mostly weak (P1: 0.18±0.31; P2: −0.01±0.06), whereas maternal genetic correlations were moderate and positive (P1: 0.39±0.95; P2: 0.46±0.04). Although the genetic influence of GL on CE was not entirely clear due to large estimation errors for parameters, overall positive associations between direct effects and maternal effects essentially indicate a selection potential for GL as an indicator trait of CE.

**Conclusion:**

This is the first genetic correlation investigation of GL and CE in Korean Holstein cattle. It provides important insights into genetic architectures of GL and its future potential as an indicator trait for CE improvements in Korean Holsteins.

## INTRODUCTION

Calving ease (CE) is a reproductive trait in dairy cattle that indicates the amount of assistance rendered during a calving event of dam. Generally, the higher the difficulty at calving (or calf-birth), the greater the phenotypic measure. CE has gained attention from dairy researchers in the past few decades due to increased concerns for farm production costs and other animal health issues in cows and calves. Many reports have suggested critical issues such as loss of calf and dam, increased labor and veterinary fees, and other long-term aftereffects such as cow fertility, digestive disorders, production cuts, and higher culling rates in farms [1,2]. All these reports emphasize the necessity for genetic improvement of CE in dairy cattle.

CE evaluation is complex for several reasons. First, both progeny and dam simultaneously contribute to the same CE phenotype. Genetic effects of CE are considered to be direct and of maternal origins [3]. A direct genetic component indicates a calf’s ability to be born easily, whereas a maternal genetic component explains the ability of the cow to give birth easily. Biological aspects of both effects have been explained in other studies [4]. Generally, a negative association between direct and maternal effects is likely. As Meijering [4] suggested, female calves are more likely to be born easily due to their smaller sizes than male calves. Female calves are also at higher odds of experiencing calving difficulties as dams due to their smaller pelvic dimensions. In contrast, a male calf is more likely to experience calving difficulty due to its higher birth weight than a female calf. Consequently, selection for direct genetic effect of CE would likely negatively impact the other, causing less efficiency in genetic improvement programs.

Gestation length (GL) in cattle is often indirectly related to CE due to its direct association with calf weight [5]. GL is a moderately heritable trait and often highly heritable than CE. A longer GL can lead to higher birth weight, subsequently increasing risks of undesired calving [4]. This argument was further strengthened by a study of Johanson and Berger [6], which listed a 13% increase in the odds of CE for a 1 kg increase in calf’s birth weight. A shorter GL can complicate calving events through fetal mortality, twins, and premature calving [4]. Nonetheless, GL can also be influenced by factors such as dam parity, calf (fetal) gender, breed of sire or dam, and other environmental factors. Some studies have also suggested evidence of associations between high CE and short GL (<265 days) and long GL (>285 days) in dairy heifers [7]. Dairy cows carrying male calves were also found to have longer GL [8]. A few studies also have identified GL as a characteristic of fetus than of dams [9,10]. This was later supported by Norman et al [11] based on the higher inheritance of service sire effect than those on cow sire effects. Therefore, understanding GL for CE evaluation becomes crucial, given that GL is more heritable than CE. Investigating both traits together as calf traits could also help better understand Holstein’s calves’ genetic potential for improving CE in the Korean dairy cattle population.

For Korean Holstein, only a few reports are available regarding their genetic potential for CE and GL [12,13]. There is no report on genetic associations of these traits in Korean Holsteins. Thus, the objective of this study was to assess the genetic merit of direct and maternal effects of CE and GL and their genetic correlation using an a multiple-trait animal model with maternal effects.

## MATERIALS AND METHODS

### Animals, phenotype, and pedigree data

CE and GL traits of the first and second-parity Holstein calves born between 2002 and 2024 in South Korea were analyzed. The Dairy Cattle Improvement Center (DCIC), National Agricultural Cooperative Federation (NACF), Korea provided all phenotypic records. CE and GL were defined as phenotypes of the progeny. This definition strategy enabled the detection of a sire’s direct contribution to calves causing CE and GL. CE scores spanned from 1 to 4: CE of 1 (non-assisted calving, no help rendered), CE of 2 (slightly assisted calving, assisted by one person), CE of 3 (moderately assisted calving, assisted by two or more persons), and CE of 4 (difficult calving, veterinary assistance required). An increment in the CE score indicated an increased difficulty at calf-birth. Therefore, a higher assistance was rendered to a dam while greater difficulties during a birth event. The range for the GL related to a calf was constrained between 260 and 305 days. Using pedigree and birth information of animals and calving information of dams, we identified valid calf information as possible. If a calf had no valid identification but valid parental information, we assigned an imaginary ID to include them for genetic analysis. Four calving seasons (Spring: March to May; Summer: June to August; Autumn: September to November; Winter: December to February) were considered.

Furthermore, we applied a series of restrictions to raw datasets, including allowing calves with all parents’ information and those unrelated to multiple births (twins and triplets). The age of the dam (at parturition) of a calf was restricted to 20 to 42 months and 30 to 54 months for parity 1 and 2 datasets, respectively. To minimize any unintended recording bias by farmers, we also removed all records of farms that provided only normal calving (CE of 1) phenotype. Finally, we constrained all datasets for a minimum of five CE observations per herd-year (HY) level and prepared two datasets parity-wise. After data pruning, parity-1 and parity-2 datasets included 117,921 and 141,104 calves for statistical analysis. All pruned datasets (see Table 1) comprised information on the calf’s sex (SEX), calf’s birth herd, birth year, birth season, and dam’s calving age (DCA; in days). We prepared related animal pedigree files for each dataset from a pedigree database provided by the Korea Animal Improvement Association (KAIA). The pedigree for individual parity datasets comprised 317,126 and 375,519 animals traced back to 23 generations.

### Statistical analysis

We implemented a bivariate animal model with maternal effect for individual parity dataset analysis to estimate (co)variance components and genetic parameters for CE and GL. The statistical model for all traits was the same. The model included two random genetic components, i.e., a direct effect of individual animals (or calves) and a maternal effect of dams. Both direct and maternal random genetic effects were also assumed to be correlated. The effect of SEX was a fixed effect, whereas that of DCA (in days) was a fixed covariate effect. Our animal model also included a composite birth herd-year-season (HYS) as a fixed contemporary group effect, which combines the calf’s birth herd, birth year, and birth season effects. The BLUPF90+ software package was used to estimate variance components, genetic parameters, and their standard errors (SEs) [14]. The linear mixed model of the studied animal model with maternal effect in matrix notation was as follows:


(Eq. 1) $$\text{y}=X\text{b}+Z_{\text{d}}\text{d}+Z_{\text{m}}\text{m}+e$$

where y was the vector related to CE and GL observations; b was the vector of fixed effects, i.e., SEX, DCA, HYS; d was the vector of random animal (direct) effect; m was the vector of random maternal effect; and e was the vector of random residual effect. **X**, **W**, **Z**_d_, and **Z**_m_ were design matrices relating effects to phenotypes.

A symmetric covariance matrix structure for random effects was assumed as follows (lower triangle is given only):


(Eq. 2) $$\text{var}\left[\begin{array}{c} d_{1}\\ m_{1}\\ d_{2}\\ m_{2}\\ e_{1}\\ e_{2} \end{array}\right]=\left[\begin{array}{cccccc} A\sigma_{d_{1}}^{2} & \cdot & \cdot & \cdot & \cdot & \cdot \\ A\sigma_{m_{1}d_{1}} & A\sigma_{m_{1}}^{2} & \cdot & \text{symmetric} & \cdot & \cdot \\ A\sigma_{d_{2}d_{1}} & A\sigma_{d_{2}d_{1}} & A\sigma_{d_{2}}^{2} & \cdot & \cdot & \cdot \\ A\sigma_{m_{2}d_{1}} & A\sigma_{m_{2}m_{1}} & A\sigma_{m_{2}d_{2}} & A\sigma_{m_{2}}^{2} & \cdot & \cdot \\ 0 & 0 & 0 & 0 & I\sigma_{e_{1}}^{2} & \cdot \\ 0 & 0 & 0 & 0 & I\sigma_{e_{1}e_{2}} & {I\;}_{e_{2}}^{2} \end{array}\right]$$

where $\sigma_{d_{i}}^{2}$ was the direct genetic variance, $\sigma_{m_{i}}^{2}$ was the maternal genetic variance, $\sigma_{e_{i}}^{2}$ was the residual variance, ***σ****_d_*_*_i_*_*_m_*_*_i_*_ was the covariance between direct and maternal genetic effects for trait *i* (*i* = 1 for trait 1, and *i* = 2 for trait 2). The **A** and **I** terms denoted the additive relationship matrix and identity matrix, respectively. Therefore, the genetic covariance matrix (**G_0_**) between *d* and *m* for the bivariate analysis was:


$${\text{G}}_{0}=\left[\begin{array}{cccc} \sigma_{d_{1}}^{2} & \sigma_{d_{1}m_{1}} & \sigma_{d_{1}d_{2}} & \sigma_{d_{1}m_{2}}\\ \sigma_{m_{1}d_{1}} & \sigma_{m_{1}}^{2} & \sigma_{m_{1}d_{2}} & \sigma_{m_{1}m_{2}}\\ \sigma_{d_{2}d_{1}} & \sigma_{d_{2}m_{1}} & \sigma_{d_{2}}^{2} & \sigma_{d_{2}m_{2}}\\ \sigma_{m_{2}d_{1}} & \sigma_{m_{2}m_{1}} & \sigma_{m_{2}d_{2}} & \sigma_{m_{2}}^{2} \end{array}\right]$$

Total phenotypic variance ( $\sigma_{p_{i}}^{2}$) [16], different heritability estimates (direct- ${\text{h}}_{d_{i}}^{2}$, maternal- ${\text{h}}_{m_{i}}^{2}$), and direct-maternal genetic correlation (**r***_d_*_*_i_*_*_m_*_*_i_*_) calculated by the above (co)variance component estimates of direct and maternal genetic effects for trait *i* (*i* = 1 for trait 1, and *i* = 2 for trait 2) are shown as follows:


$$\begin{array}{l} \sigma_{p_{i}}^{2}=\sigma_{d_{i}}^{2}+\sigma_{m_{i}}^{2}+\sigma_{e_{i}}^{2}+\sigma_{d_{i}m_{i}}\\ {\text{h}}_{d_{i}}^{2}=\frac{\sigma_{d_{i}}^{2}}{\sigma_{{\text{p}}_{i}}^{2}}\\ {\text{h}}_{m_{i}}^{2}=\frac{\sigma_{m_{i}}^{2}}{\sigma_{{\text{p}}_{i}}^{2}}\;\;\;,\text{and}\\ {\text{r}}_{d_{i}m_{i}}=\frac{\sigma_{d_{i}m_{i}}}{\sqrt{\sigma_{d_{i}}^{2}\times \sigma_{m_{i}}^{2}}} \end{array}$$

Between-trait direct and maternal effect correlations were also calculated using respective (co)variance components of the two traits. We additionally analyzed both parity-wise datasets using a second bivariate animal model without fitting the maternal effect to understand the significance of maternal effects for each trait. Genetic parameters for traits using the latter model were calculated as ${\text{h}}_{d_{i}}^{2}=\frac{\sigma_{d_{i}}^{2}}{\sigma_{p_{i}}^{2}}$, where $\sigma_{d_{i}}^{2}$ was the additive genetic variance and $\sigma_{p_{i}}^{2}$ (the phenotypic variance) was the sum of $\sigma_{d_{i}}^{2}$ and $\sigma_{e_{i}}^{2}$ ( $\sigma_{e_{i}}^{2}$ was the residual variance for trait *i*). Genetic correlation between the two traits was also calculated as $r_{g}=\frac{\sigma_{d1d2}}{\sqrt{\sigma_{d1}^{2}\times \sigma_{d2}^{2}}}$, where σ*_d_*_1_*_d_*_2_ was the genetic covariance between the two traits.

Approximated SE of genetic parameters were obtained from (co)variance components using the BLUPF90+ software package, in which a Monte Carlo method was implemented for the computation of SE following a previous study [16]. Each individual’s expected progeny difference (EPD) was calculated based on direct and maternal effects solutions from BLUPF90+ software analysis as (H_sire_+H_dam_)/2, where H_sire_ and H_dam_ were total performance of all progenies of its sire and dam, respectively. A correlation on EPDs across parity records was calculated using commonly available animals within two pedigree data sets. We further obtained estimated breeding value (EBV) trends for direct and maternal genetic components of animals after normalization of EBV estimates using Z-statistic to facilitate plotting of two traits EBVs of different scales into one figure.

## RESULTS

### Descriptive statistics and phenotypic trends

**Figure 1:**
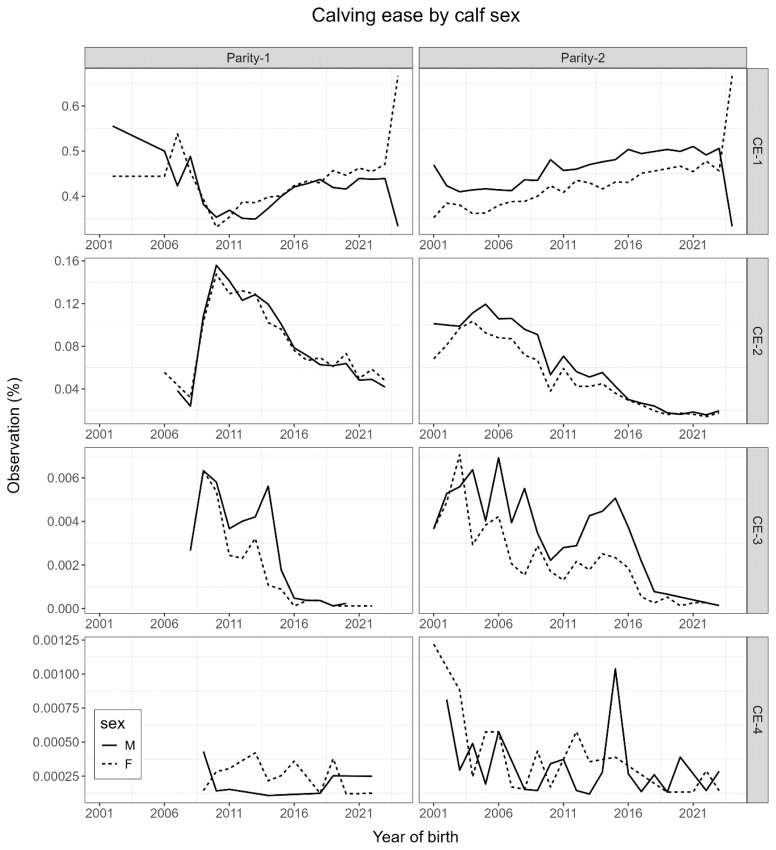
Trend of four calving ease categories within males and females (CE-1: non-assisted calving; CE-2, slightly assisted calving; CE-3, moderately assisted calving; CE-4, difficult calving with veterinary assistance) born as parity 1 and 2 crops of Korean Holstein. CE, calving ease.

**Figure 2:**
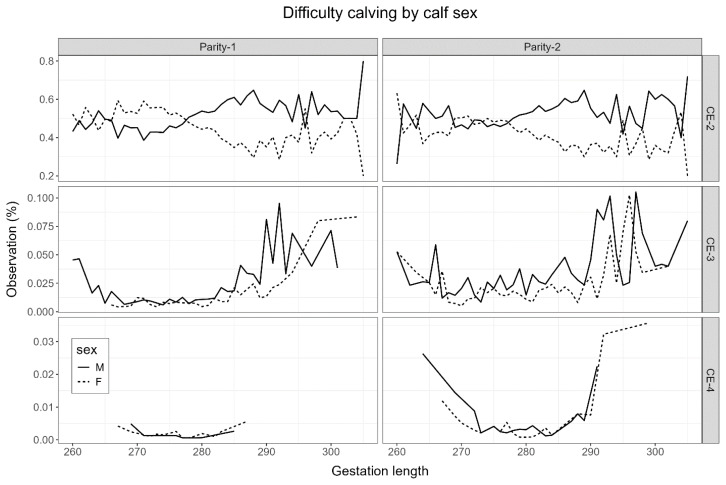
Trend of difficulty calving incidences within males and females (CE-2, slightly assisted calving; CE-3, moderately assisted calving; CE-4, difficult calving with veterinary assistance) born as parity 1 and 2 crops. CE, calving ease.

Table 1 presents overall CE and GL phenotype statistics relevant to calves born in their dam’s first and second parities. In this study, a large proportion (82% to 89%) of calves had unassisted normal births across parities (Figure 1). GL on average was about 1.8 days longer with the second parity calves, where the first parity average GL was about 277 days. Female calves also had an average GL shorter by a day than male calves. GL varied slightly within CE categories. The phenotypic trend of CE in this study also showed an association between females and easy calving and the most extreme calving events (Figure 1). However, male calves had more incline towards low and moderate calving difficulties at birth. The distribution of CE and GL further indicated greater odds for calves requiring more assistance when GL was about 285 days or more (Figure 2). Figure 2 also indicated higher odds for female progenies with shorter GL. All Korean Holstein calves had greater odds of extreme difficulties with short and long GL.

### Genetic parameter estimates and genetic trends

For the estimation of genetic parameters, parity-wise datasets were analyzed using two bivariate animal models: with or without maternal effects. Tables 2 to 4 present (co)variance components of genetic effects and genetic parameters estimates of genetic effects from bivariate animal model analyses using P1 and P2 datasets. Results showed that both direct CE (DCE) and maternal CE (MCE) were lowly heritable (~0.01) in all parity calves. For direct GL (DGL), h^2^ estimates were between 0.20 and 0.23. Interestingly, the maternal GL (MGL) effect had a relatively low h^2^ range (~0.03) in all parity calves. Without fitting the maternal effect in the second animal model, h^2^ estimates of DCE and DGL were somewhat similar to those from the model that included the maternal effect.

Trait-wise genetic associations between direct and maternal effects across parity also varied widely (Table 3). Between DCE and MCE, the genetic correlation estimate appeared slightly positive (P1: 0.09±16.61) or lowly negative (P2: −0.04±0.00), indicating a weak association. The large SE of estimates also indicated some possible estimation bias. GL also showed a similar low association between direct and maternal effects (P1: 0.03±0.00; −0.15±0.05). Between DGL and DCE, we observed weaker genetic correlations in two parity records (P1: 0.18±0.31; P2: −0.01±0.06), which were in contrast to the moderately positive genetic correlation between MGL and MCE effects (P1: 0.39±0.95; P2: 0.46±0.04). These correlation estimates were not significantly different from zero due to a low precision. The model without maternal effects also showed positive genetic correlations between GL and CE (Table 4). However, positive correlations, especially between MGL and MCE effects, indicated positive influences on CE if selection was performed for an optimal GL.

**Figure 3:**
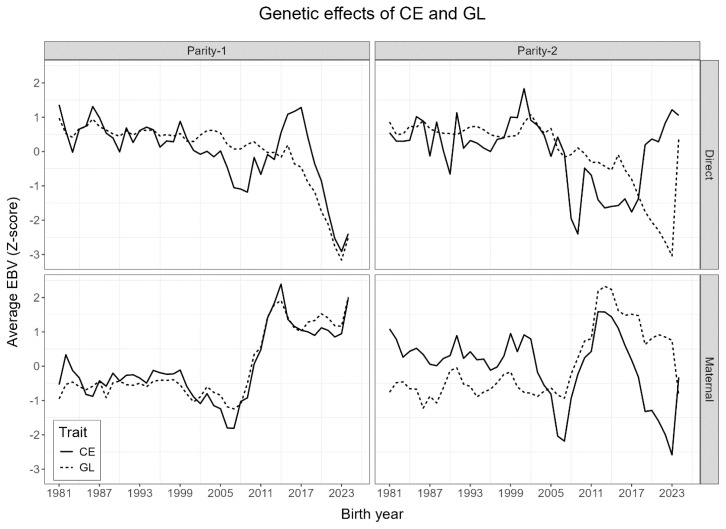
Trend of average of normalized estimated breeding values (EBVs) for direct and maternal calving ease (CE) and gestation length (GL) using all animals in pedigree related to the first and second parity Korean Holstein datasets.

Genetic trends of average (normalized) direct and maternal EBVs for all animals in the pedigree are plotted in Figure 3. Overall, both traits’ genetic effects showed similar trends over time. The trend was consistent for direct and maternal genetic effects up to 2005. Such consistency was mainly to the fact that those animal EBVs were based on parental averages due to an absence of actual phenotypic data for that whole period. Animals showed an overall increase in direct EBVs from 2008 to 2017, indicating an increased calving difficulty in the population, although direct EBV of GL decreased simultaneously. With maternal EBVs, both traits showed positive average increases in both traits, which could also explain their moderate correlation. The later trend inconsistencies could be mainly due to smaller sample sizes from those years.

Table 5 presents correlation estimates between EPDs for each genetic effect between P1 and P2 calves. As animals with phenotypes were unique to each parity dataset, these correlations were based on shared animals (e.g., primarily parents) related to the pedigree of the two datasets. EPD correlations were moderately positive with GL and relatively weak with CE. Rank correlation estimates were also similar to those of Pearson’s correlations. This lower magnitude in relationships for the similar genetic effect, especially the lower rank correlations, indicates that animals’ ranking for each trait would differ across parity. These estimates are not a unity (i.e., 1.0), suggesting that CE and GL defined over different parities might not be the same trait in genetic terms. Therefore, evaluating parity-wise phenotype datasets may help optimize selection outcomes for the overall population. The present study also included the Akaike information criterion (AIC) statistic between models with and without maternal effects. The model that included maternal effects had a smaller AIC value, suggesting the importance of maternal effects in the model. Therefore, including maternal genetic effect in the animal model deemed appropriate for evaluating these reproduction traits.

## DISCUSSION

Many reports have suggested associations among calf sex, parity, GL, and CE. Regarding calf sex, one study by Johanson and Berger [6] has shown a 25% higher odds for assistance required for males than for females. According to many reports, GL is increased with an increase of lactation number [5,17]. Studies of Philipsson [7] and Atashi and Asaadi [18] have indicated a positive association between longer GL and increased difficulty calving due to higher birth weights with longer GLs. In addition to longer GL, shorter GL also contributes to greater calving difficulties based on a study of Nogalski and Piwczyński [5] on Polish Holstein-Friesian cows. Our report found that an intermediate GL had the lowest occurrences of difficult calving observations. Multiple Holstein cattle studies have also indicated potential benefits of intermediate GLs on CE and other production performances in dairy cattle [11,19].

Further note that CE in dairy cattle is often treated as a trait of the calf rather than the dam, which offers several advantages for the genetic evaluation of animals. CE as a calf trait allows calves of both sexes from the current generation to contribute to the genetic evaluation process based on the calf’s morphological features (i.e., calf birth weight and shapes). This approach allows for the direct estimation of service sire’s genetic contribution to CE in its offspring, which is also crucial for selecting bulls in dairy cattle. In contrast, CE as a dam’s trait allows fewer animals (i.e., primarily dams) to be evaluated, and the following evaluation is based on the dam’s pelvic dimension and other maternal characteristics. Similar to CE, the GL has also been considered a calf trait in the literature, mainly when analyzed together with the former trait [20,21]. The justification for such consideration is explained in the introduction section of this article.

Genetic merits of direct and maternal effects of CE have been widely reported in the literature. Many studies have considered CE either as a calf trait or a dam trait. In this study, we defined it as a calf trait. We observed that both DCE and MCE effects were lowly heritable (less than 1%), even lower than our previously reported estimates using an sire-maternal grandsire (S-MGS) model (direct h^2^: 0.11 and maternal h^2^: 0.05) in first parity records [13]. Previously, Eaglen and Bijma [16] and Mujibi and Crews [22] have also shown lower h^2^ estimates but slightly higher than ours. Our report agreed with an animal model study on Iranian Holstein cattle [23] suggesting lower h^2^ estimates (direct: 0.041, maternal: 0.012). The report of Salimi et al [24] on Iranian Holstein also closely corroborates our estimates. Eaglen et al [25] have reported similar low heritability estimates for DCE (0.03) and MCE (0.02). Generally, a lowly heritable direct effect is available from many dairy cattle reports in the last few decades [26–28]. Our estimates for direct and maternal effects could also corroborate with those reported by others [29,30]. Some beef cattle studies, such as a study by Roughsedge et al [31], also demonstrated that despite low maternal h^2^ across beef breeds, their direct h^2^ could vary widely (0.13 to 0.35).

Both Ibi et al [20] and Inoue et al [21] have estimated direct h^2^ (0.53) and maternal h^2^ (0.14), considering GL as a calf trait in Japanese Black cattle. Their h^2^ estimates were slightly higher than our estimates. Mujibi and Crews [22] have also reported higher estimates in Charolais cattle (direct h^2^: 0.62 and maternal: 0.14). On the other hand, slightly lower h^2^ estimates for DGL and MGL (0.27 to 0.43 and 0.07 to 0.13, respectively) were reported for Holstein [32,33]. MGL h^2^ estimates in earlier reports were as low as 0.06 to 0.18 [7,34]. Our DGL and MGL h^2^ estimates were also close to those reported by these authors. Some closer agreements were also found in other dairy cattle reports [35–37], where their values were within our range (direct: 0.27 to 0.42; maternal: 0.04 to 0.13). Several studies have found that genetic variation of GL is large enough to change GL through selection [17,32,33]. These moderate to high values of direct heritability suggest genetic progress could be made for GL if this trait is considered in genetic evaluations and included in selection indices.

On the contrary, we found relatively weaker positive and negative correlations between direct (DGL vs. DCE) and maternal effects (MGL vs. MCE). Similar weakly negative to positive correlations have also been found in Japanese Black cattle [21], i.e., genetic correlations of 0.32 (direct) and −0.19 (maternal). However, our maternal h^2^ estimates were slightly higher than theirs. Genetic correlations between DGL and DCE (0.38) and between MGL and MCE (0.18) in Danish Holsteins [33] were not much different from our estimates, despite an opposite pattern of estimates in our values. Our estimates were also within the range of estimates reported by de Maturana et al [38]. Mujibi and Crews [22] have also demonstrated that genetic effects of GL and CE are slightly negatively correlated (DGL vs. DCE: −0.38, MGL vs. MCE: −0.49). A weak to strong genetic correlation range between genetic effects of GL and CE traits is also observed across reports [33,34]. Studies not accounted for maternal effects have also reported very weak to moderate positive genetic correlations between DGL and DCE [39].

This study investigated the genetic relationships between GL and CE, focusing on the calf’s genetic potential for direct (DGL, DCE) and maternal (MGL, MCE) effects. Overall, a weak correlation between these traits provided no strong evidence for a potential negative influence during the selection of animals for both traits. A weak association between DGL and DCE (i.e., 0.18 in parity 1 calves) could eventually benefit the breeding program as the selection of sires for reduced calf size and shapes (via selection for low DCE-EBVs) will have a lesser impact on DGL-EBVs and vice versa. Note that an increase in DGL-EBVs (i.e., longer GL) could further complicate calf births. Similarly, selection for a higher MCE EBVs, which indicates an improvement in the female calf’s (or future dam’s) pelvic dimension, would not strongly affect the calf’s future maternal ability for GL due to weak MGL and MCE relationship. Generally, GL influences are nonlinear on CE, i.e., intermediate GLs cause desired CE scores, whereas the lower and higher extremes of GL cause undesired CEs. Therefore, GL’s lack of strong correlation with CE would help optimize both traits while having minimal genetic interference on calves. Some reports [40] have suggested that GL is more appropriate as an indicator trait for the lowly heritable CE than a selection trait. Also, a direct selection progress of GL without references to CE or other important dairy traits could jeopardize the overall selection progress for CE in dairy production [40].

On the other hand, genetic correlation estimates in this report suffered in precision, causing inconsistencies in genetic parameter estimates across parities. The analyzed datasets were limited to fewer undesired CE scores than desired ones, which might also contribute to such estimation inconsistencies. Further investigation into the dataset revealed a possible nested structure between animals and herds as dams and their progenies hardly changed herds. This could lead to an inefficient separation of genetic effects from environmental components. Therefore, using an S-MGS model might help overcome data structure limitations and improve the quality of genetic parameters. Considering CE and GL as dam traits can also help compare and better understand the present outcomes. Further research with more data, redefining phenotypes, and using an S-MGS model in Korean Holstein is needed for further understanding.

## CONCLUSION

This study investigated the genetic correlation of direct and maternal effects of GL and CE on first and second parity Korean Holstein progenies. Two bivariate animal models with and without maternal effects were utilized. The DGL effect was moderately heritable; MGL and all effects of CE were lowly heritable. Direct and maternal genetic correlations between GL and CE were primarily low, except for a few moderate relationships. The influence of GL on CE was not realized clearly due to low precisions for genetic parameters. The lack of precision is attributed to the inefficiency of the animal model adjusting for existing data structures (e.g., fewer samples per HYS, females being nested to herds). However, GL’s moderate direct heritability and its non-antagonistic relationships with CE via all genetic paths provide future selection prospects, especially for using GL as an indicator of CE. Selecting GL for an intermediate optimum range to improve CE could be a viable option for future breeding initiatives in Korea. Also, being the first Korean Holstein report regarding GL and CE genetic association, present estimates could provide valuable insights into future dairy cattle investigations.

## Figures and Tables

**Table 1:** The structure of phenotype datasets of calves born in first and second parities of Korean Holstein cows

Factor/Term	Level	Parity-1	Parity-2
Number of observations	-	117,921	141,104
Calf-sex	Male	58,170	74,403
	Female	59,751	66,701
Birth herd (H)	-	1,394	2,154
Birth year (Y)	-	2002–2024	2001–2024
Birth season (S)	Spring	30,000	31,401
	Summer	28,611	33,123
	Autumn	29,351	40,397
	Winter	29,959	36,183
Number of HYS	-	37,226	57,939
Number of Sires	-	1756	2002
Number of Dams	-	117,921	141,104
Gestation length (GL, mean±SD)	-	277.1±5.7	278.9±5.9
Calving ease (CE)	1	96,611	126,376
	2	20,856	13,998
	3	418	659
	4	36	71
Calf-sex by GL (mean)	Male	277.6	279.4
	Female	276.6	278.4
CE by GL (mean)	1	277.1	278.9
	2	277.1	279.6
	3	278.9	280.9
	4	276.4	279.4

SD, standard deviation.

**Table 2:** Estimates of (co)variance components of random genetic effects and their genetic parameter estimates using a bivariate animal model^1)^

Parity	Trait^2)^	$\sigma_{\text{d}}^{2}$	σ_dm_	$\sigma_{\text{m}}^{2}$	$\sigma_{\text{e}}^{2}$	$\sigma_{\text{p}}^{2}$	${\text{h}}_{\text{d}}^{2}+\text{SE}$	${\text{h}}_{\text{m}}^{2}+\text{SE}$	AIC
First	CE	0.0003	0.0000	0.0001	0.0618	0.0623	0.005±0.003	0.001±0.002	−161198
	GL	6.8543	0.0559	0.6072	21.7330	29.2500	0.234±0.017	0.021±0.004	
Second	CE	0.0001	−0.0000	0.0003	0.0587	0.0591	0.002±0.000	0.005±0.000	−240691
	GL	6.3684	−0.3709	0.9493	24.6840	31.6310	0.201±0.011	0.030±0.002	

1) Phenotypes were considered as calf traits.

2) Variance estimate of (±)0.0000 indicates value smaller than (±)0.00004.

CE, calving ease; GL, gestation length; $\sigma_{\text{d}}^{2}$, direct genetic variance; σ_dm_, covariance between direct and maternal genetic variance; $\sigma_{\text{e}}^{2}$, residual variance; $\sigma_{\text{p}}^{2}$, phenotypic variance; ${\text{h}}_{\text{d}}^{2}$, direct heritability estimate; ${\text{h}}_{\text{m}}^{2}$, maternal heritability estimate; AIC, Akaike information criterion.

**Table 3:** Estimates of genetic correlations between direct and maternal effects using bivariate animal model with maternal effect

Parity/Trait		DCE	DGL	MCE	MGL
First	DCE	-	0.18±0.31	0.09±16.61	−0.02±0.38
	DGL		-	−0.04±0.66	0.03±0.00
	MCE			-	0.39±0.95
	MGL				-
Second	DCE	-	−0.01±0.06	−0.04±0.00	−0.14±0.06
	DGL		-	0.26±0.05	−0.15±0.05
	MCE			-	0.46±0.04
	MGL				-

DCE, direct calving ease; DGL, direct gestation length; MCE, maternal calving ease; MGL, maternal gestation length.

**Table 4:** Estimates of variance components, and genetic parameters using a bivariate animal model without maternal effect^1)^

Parity	Trait^2)^	$\sigma_{\text{d}}^{2}$	$\sigma_{\text{e}}^{2}$	$\sigma_{\text{p}}^{2}$	h^2^±SE	r_g_	AIC
First	CE	0.0001	0.0621	0.0622	0.016	0.32	422340
	GL	7.7827	21.1870	28.9690	0.269		
Second	CE	0.0001	0.0589	0.0591	0.002	0.07	450080
	GL	5.7396	25.4210	31.1610	0.184		

1) Phenotypes were considered as calf traits.

2) Variance estimate of (±)0.0000 indicates value smaller than (±)0.00004.

$\sigma_{\text{d}}^{2}$, additive genetic variance; $\sigma_{\text{e}}^{2}$, residual variance; $\sigma_{\text{p}}^{2}$, phenotypic variance; h^2^, heritability estimate; r_g_, genetic correlation estimate between CE and GL; AIC, Akaike information criterion; CE, calving ease; GL, gestation length.

**Table 5:** Pearson’s correlation and Spearman’s rank correlation (within parentheses) for direct and maternal expected progeny difference (EPD) in Korean Holsteins

Trait^1)^	EPD-direct (P1 vs. P2)	EPD-maternal (P1 vs. P2)
CE	0.09 (0.08)	0.27 (0.19)
GL	0.46 (0.46)	0.37 (0.38)

1) Phenotypes were considered as calf traits; Animals’ existing in both parity pedigree datasets were used for correlation estimates.

P1, first parity; P2, second parity; GL; gestation length; CE, calving ease.
